# Monitoring juvenile sicklefin lemon shark *Negaprion acutidens* in remote marine nurseries using unmanned aerial vehicles (UAVs)

**DOI:** 10.1038/s41598-025-21142-y

**Published:** 2025-10-24

**Authors:** Chia-Yun Joanne Li, Chin-Ti Lin, Keryea Soong

**Affiliations:** 1 Coastal and Offshore Resources Research Center, Fisheries Research Institute, Ministry of Agriculture, Kaohsiung, 80672 Taiwan; 2 Department of Research and Conservation, Georgia Aquarium, Atlanta, GA 30313 USA; 3 Department of Oceanography, National Sun Yat-sen University, Kaohsiung, 804201 Taiwan

**Keywords:** *Negaprion acutidens*, Spatial ecology, Drone survey, Anthropogenic disturbance, Dongsha atoll, UAV, Ecology, Marine biology

## Abstract

Understanding spatial and demographic patterns in threatened coastal sharks is essential for effective conservation, yet remote reef systems remain understudied due to logistical constraints. We used dual unmanned aerial vehicles (UAVs) to monitor juvenile *Negaprion acutidens* around Dongsha Atoll, a no-take marine reserve in the northern South China Sea. Thirteen synchronized UAV surveys were conducted during summer and winter, covering 20 locations categorized into three zones representing different levels of human impact. We quantified seasonal variation in shark abundance, body size, spatial distribution, and environmental drivers using Generalized Linear Mixed Models (GLMMs). Results revealed stable overall abundance but strong fine-scale shifts between summer and winter. Northeastern sites showed sharp declines in shark sightings during winter, likely due to monsoonal exposure, while sheltered southern zones supported increased winter presence. Neonates were concentrated in lagoon habitats, whereas larger individuals occurred farther offshore and in seagrass areas, indicating ontogenetic habitat expansion. Human impact shaped demographic structure: low-impact areas hosted more and larger sharks, while smaller individuals and nearshore aggregation dominated high-impact zones. These findings confirm Dongsha’s role as a critical nursery habitat for *N. acutidens* and highlight the utility of UAV surveys for capturing spatiotemporal ecological patterns in remote ecosystems. By integrating UAV monitoring with conservation planning, managers can prioritize seasonal protection of nursery zones and respond adaptively to climate and anthropogenic pressures.

**Supplementary Information:**

The online version contains supplementary material available at 10.1038/s41598-025-21142-y.

## Introduction

Understanding the spatial distribution of marine species is fundamental for assessing ecosystem health, guiding conservation strategies, and informing management decisions^1^. As top predators, sharks help maintain ecological balance by regulating prey populations and shaping trophic dynamics^2,3^, yet many have declined due to overfishing, habitat loss, and climate-driven changes^4,5^.

Coastal sharks, which inhabit continental shelf and nearshore waters (0–200 m depth), depend on mangroves, seagrass beds, lagoons, and estuarine environments for foraging and as critical nursery grounds^6,7^. Many exhibit strong site fidelity^8,9^, yet the habitats they depend on are under intense human pressure, resulting in over half of these species being threatened^10^. Improving our understanding of their spatial ecology, especially in ecologically sensitive regions or remote regions, is therefore critical for conservation.

Dongsha Atoll (20° 35′–47′ N, 116° 41′–55′ E), located in the northern South China Sea approximately 450 km southwest of Taiwan, lies within the Dongsha Atoll National Park (DANP), a remote, fully protected no-take marine reserve established in 2007^11^. Its shallow lagoon, fringed by seagrass beds and coral reefs, supports diverse marine life and serves as a critical nursery habitat for coastal species^12,13^. Although extractive activities are prohibited within the park, localized human presence persists year-round, including permanent military facilities, a research station, and routine vessel traffic (such as supply and patrol boats).

Juvenile *Negaprion acutidens* (sicklefin lemon shark), currently classified as Endangered by the IUCN^14^, are frequently observed in Dongsha Island and exhibit strong site fidelity^15^. Dongsha has also been proposed as a critical nursery habitat for *N.acutidens*, based on repeated sightings and records of neonates^15^. It represents a rare example of a shallow aggregation site for this species in the northern South China Sea. The local population has a notably low effective size (Ne = 86.7, Liu et al.^19^), raising additional conservation concerns given ongoing threats such as illegal fishing and habitat degradation.

Although *N. acutidens* is broadly known to inhabit tropical coastal nurseries^16–18^, studies on seasonal dynamics and spatial use remain limited, particularly in this region. Despite legal protection, anthropogenic pressures, including persistent human activity, vessel traffic, and occasional illegal fishing, remain present in the atoll, and their influence on shark distribution remains poorly understood. Existing knowledge of this population is based solely on two studies, one examining genetic structure^19^ and another on site fidelity^15^. However, fine-scale spatiotemporal patterns remain unstudied. Addressing these questions is essential for effective, evidence-based conservation planning.

Traditional monitoring tools such as diver-based surveys, mark-recapture, and baited remote underwater video systems (BRUVs) have been instrumental in improving our understanding of shark abundance, behavior, and distribution^20,21^. However, these methods can be logistically intensive and time-consuming. Unmanned aerial vehicles (UAVs) offer a rapidly deployable and scalable alternative, particularly well-suited for use in shallow, high-visibility environments^22,23^. UAVs have been successfully applied to monitor behavior, abundance, or movement patterns in several shark species, such as *Rhincodon typus*^24^, *Carcharhinus melanopterus*^25^, *Carcharodon carcharias*, *Carcharhinus leucas*, and *Carcharias taurus*^26,27^, demonstrating their utility in documenting surface aggregations, group dynamics, and spatial behavior. Compared to underwater methods, UAVs minimize observer bias and animal disturbance, require fewer field personnel, and allow efficient coverage of multiple sites in a short period, making them particularly effective for species that aggregate near the surface in remote or logistically constrained environments such as Dongsha Atoll.

In this study, we used UAV-based aerial surveys to assess seasonal variation in the abundance, body size, and spatial distribution of *N. acutidens* around Dongsha Island. By integrating habitat features and levels of human activity, we identified key ecological drivers of shark distribution and evaluated the role of marine protected areas in supporting nursery habitats. This work aims to inform conservation planning and contribute to broader efforts to apply remote sensing technologies in coastal shark monitoring.

## Results

###  UAV survey effort

**Fig. 1:**
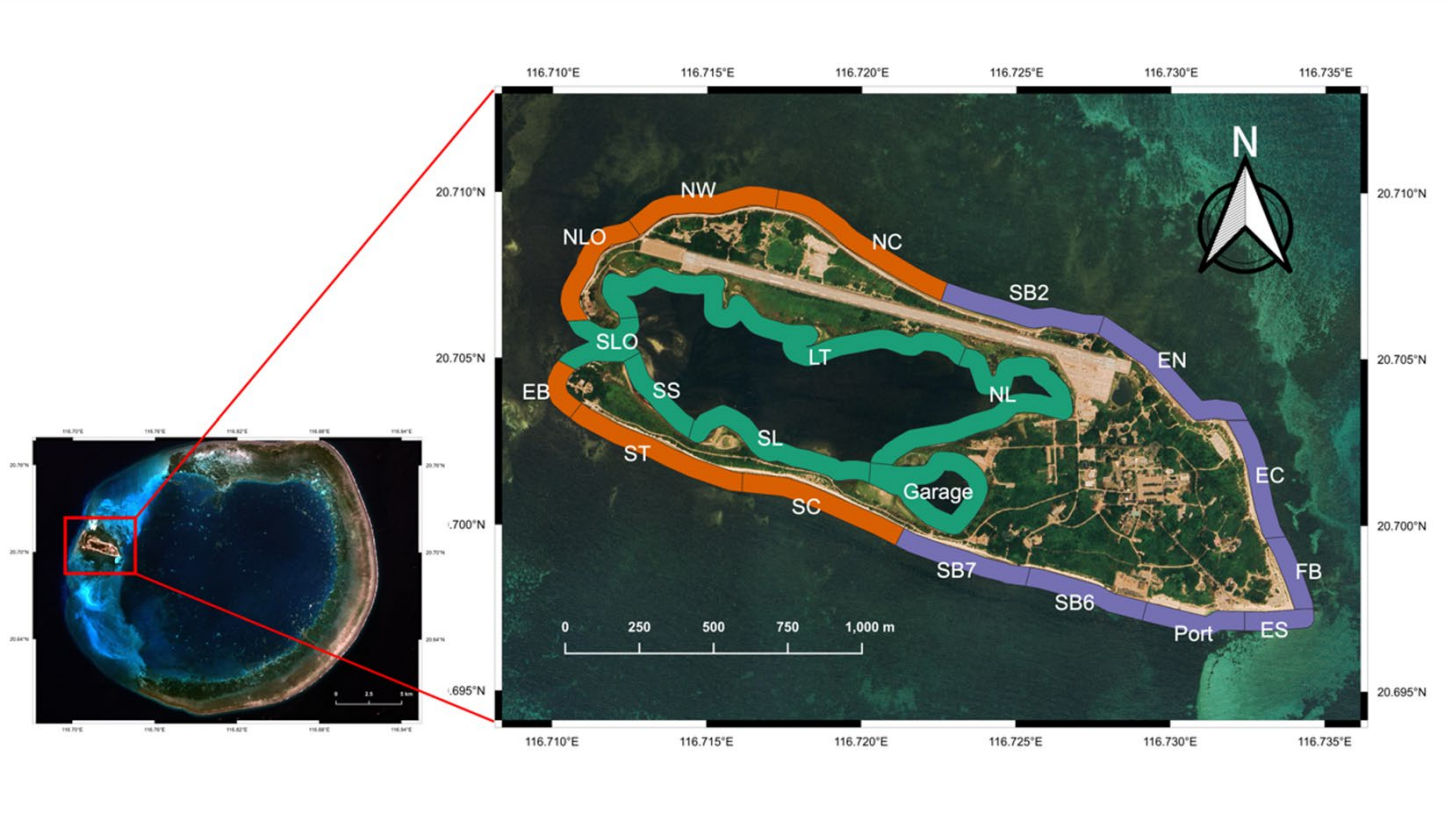
UAV transects and 20 locations around Dongsha Island. Sites are categorized as Lagoon (Green), Low-Human-Impact Coast (LHIC; Orange), High-Human-Impact Coast (HHIC; Purple).

We conducted thirteen UAV surveys spanning both lagoon and coastal habitats around Dongsha Island (Fig. 1), including six flights in August 2021 (summer) and seven in February 2022 (winter). Simultaneous UAV flights surveyed over 724,000 m^2^ of nearshore habitat in 30 min, demonstrating exceptional efficiency and spatial coverage.

### Shark abundance

#### Seasonal variation

Across all surveys, summer shark counts ranged from 56 to 157 individuals (mean ± SE: 110 ± 17.09 cm; Table S1), while winter counts ranged from 41 to 110 (mean ± SE: 71.57 ± 9.57 cm; Table S1). For analysis, the study area was subdivided into 20 locations and categorized into three habitat types based on human disturbance: Lagoon, Low-Human-Impact-Coastal areas (LHIC), and High-Human-Impact-Coastal areas (HHIC). Detailed classification is provided in the Methods section and Fig. 1.

**Fig. 2:**
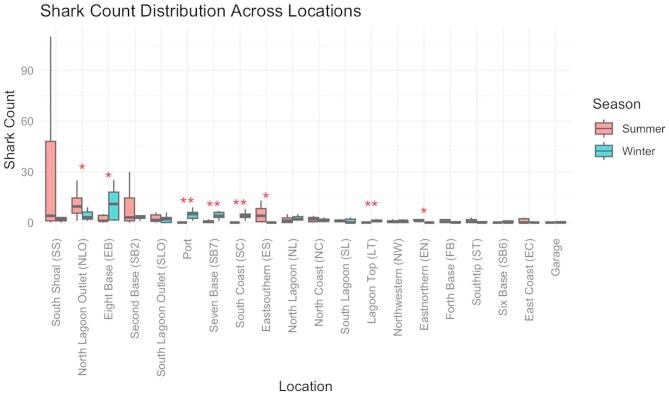
Seasonal shark abundance across sites. Asterisks: *p < 0.05, **p < 0.01.

At the site level, several locations exhibited significant seasonal shifts in abundance. In winter, shark abundance was significantly higher at SC, SB7, Port, and LT (*p* < 0.01), with a moderate increase at EB (*p* < 0.05). In contrast, Eastern sites (EN, ES) and NLO showed significantly higher abundance during summer (*p* < 0.05; Fig. 2). Other locations showed no significant seasonal differences (*p* > 0.05).

#### Environmental and Spatial drivers

The best-fit GLMM included an interaction between season and site direction, along with fixed effects of tide and substrate:

$$Shark.Count\sim Season \times Direction+Tide + Substrate + (1 | Location)$$

This model had the lowest AIC among all candidates (AIC = 1000.06; Table S2). Shark abundance was significantly higher along Lagoon (Estimate = 1.33, SE = 0.64, *p* = 0.04) and, West direction (Estimate = 2.01, SE = 0.81, *p* = 0.01) relative to the reference direction (East). Spring Tide was associated with moderately reduced counts (Estimate = −0.28, SE = 0.14, *p* = 0.04). Full model results are presented in Table 1.

**Table 1:** GLMM results for shark abundance.

Shark count ~ Season * Direction + Tide + Substrate + (1 | Location)			
Fixed effect	Estimate	Std. Error	*p* value
(Intercept)	0.90	0.71	0.20
Season			
Winter	−0.91	0.97	0.35
Direction			
Lagoon	1.33	0.64	0.04*
North	0.63	0.65	0.33
South	−0.13	0.63	0.84
West	2.01	0.81	0.01*
Tide			
Spring Tide	−0.28	0.14	0.04*
Substrate			
Sand	0.25	0.43	0.56
Seagrass	−0.80	0.43	0.06.
Interactions			
Winter × Lagoon	−0.04	1.00	0.97
Winter × North	−0.04	1.02	0.97
Winter × South	1.90	1.01	0.06.
Winter × West	−0.05	1.02	0.96

Negative binomial model with location as random intercept. Significance: *** *p* < 0.001, ** *p* < 0.01, * *p* < 0.05, *p* < 0.1.

### PCL variation

#### Seasonal and spatial patterns

Pre-Caudal Length (PCL) ranged from 40.24 cm to 191.99 cm (mean ± SE = 65.44 ± 18.10 cm), with a median of 58.08 cm. Corresponding body widths ranged from 11.41 to 62.12 cm (mean ± SE = 23.59 ± 7.16 cm) with a median of 21.62 cm. The distribution was right-skewed, indicating a predominance of smaller individuals. PCL and width were highly correlated (Pearson correlation: *r* = 0.89, Fig. S1), so PCL was used as the primary metric in subsequent analyses to simplify interpretation while preserving accuracy.

**Fig. 3:**
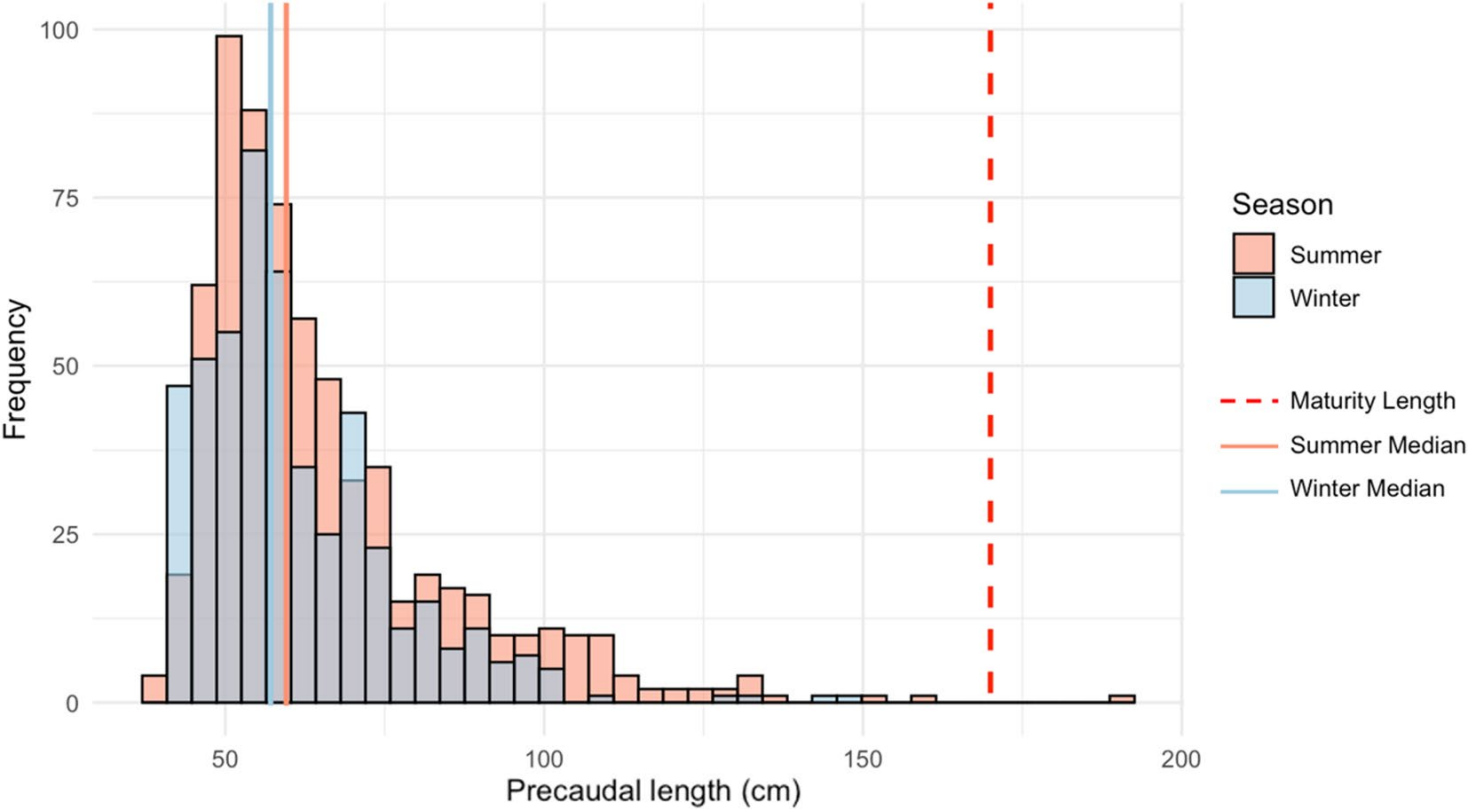
Seasonal precaudal length distribution of *N. acutidens*.

Sharks observed in winter were significantly larger than those observed in summer (Mann-Whitney U test, *p* < 0.001; Fig. 3). Spatial variation in PCL was also evident across sites (Fig. S2). Locations such as SLO (mean ± SE = 83.60 ± 21.00 cm) and SS (mean ± SE = 59.30 ± 12.20 cm) exhibited a broad size range, while sites like Garage (mean ± SE = 51.50 ± 2.76 cm), LT (mean ± SE = 53.30 ± 2.46 cm) and SB2 (mean ± SE = 55.50 ± 1.11 cm) were dominated by smaller individuals.

**Fig. 4:**
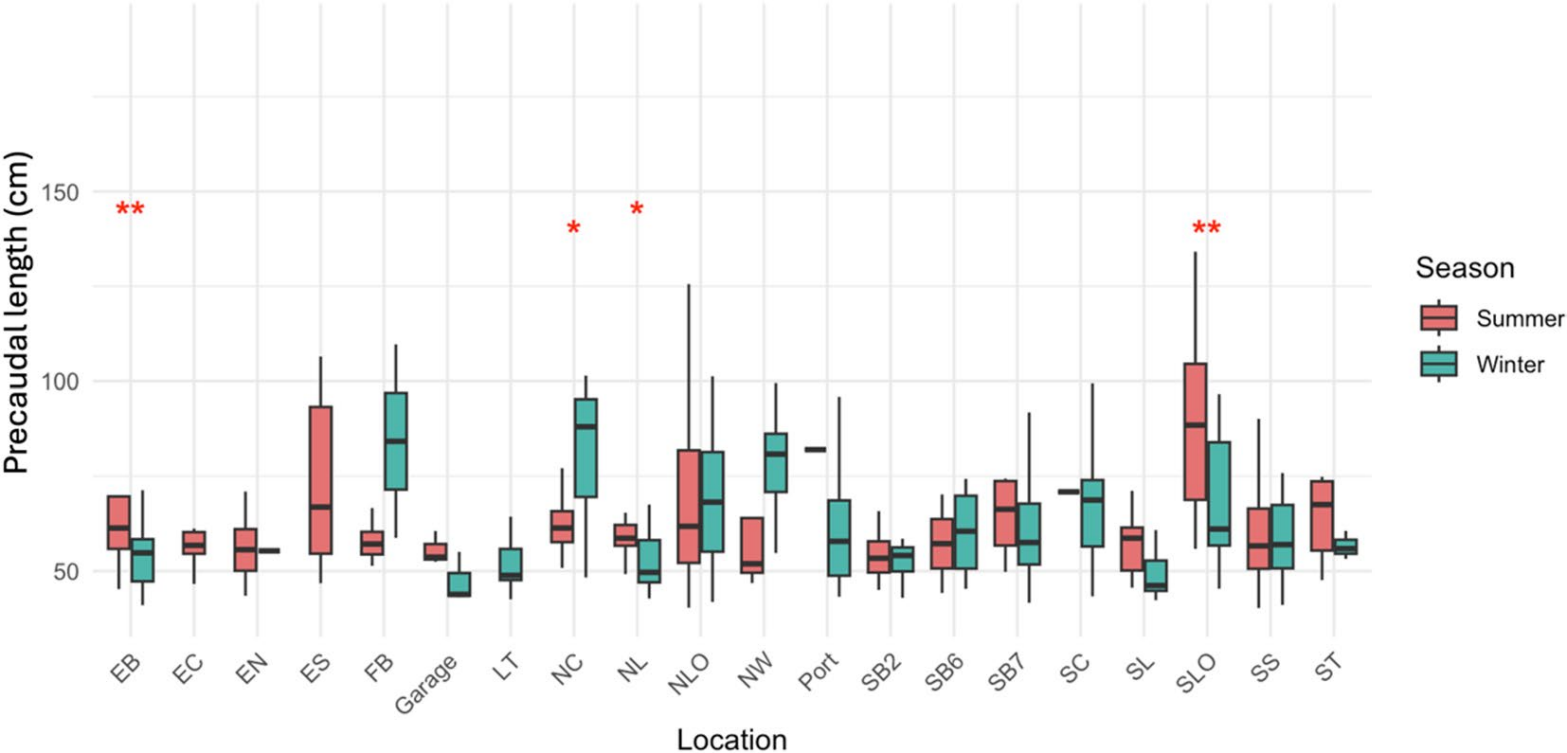
Seasonal precaudal length differences across locations. Asterisks: *p < 0.05. **p < 0.01.

Some locations also showed significant seasonal shift in size distribution. Notably, sharks at NL (*p* < 0.05), NC (*p* < 0.05), SLO (*p* < 0.01), and EB (*p* < 0.01) were significantly larger in summer than in winter (Fig. 4). The most pronounced seasonal difference occurred at SLO, where summer median PCL greatly exceeded winter values (Summer vs. Winter = 88.5:61.1).

#### Substrate and offshore distance effects

**Fig. 5:**
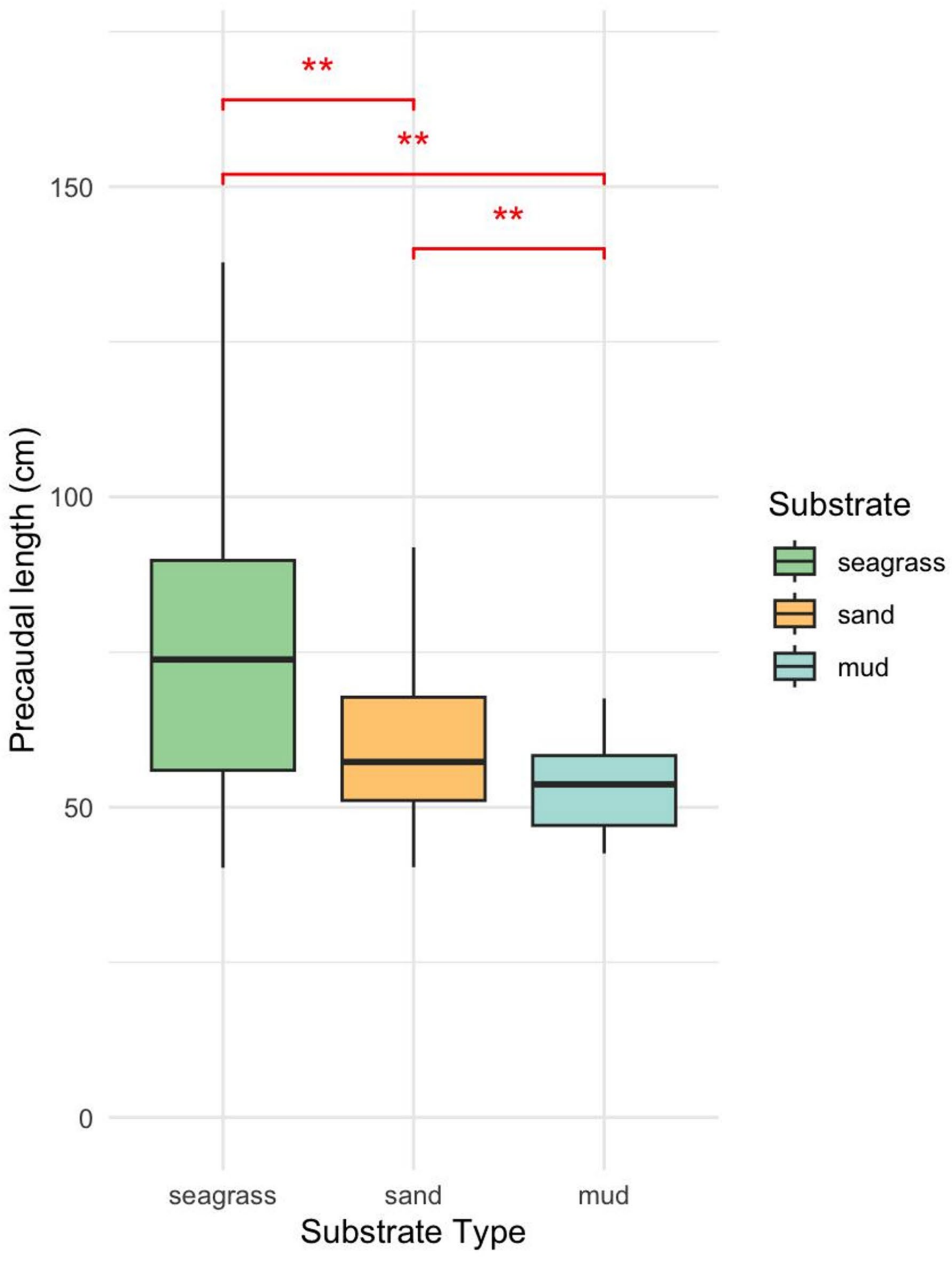
Precaudal length by substrate. Significance from Kruskal-Wallis and Dunn’s test (*p < 0.01, **p < 0.001).

PCL varied significantly among substrate types (Kruskal–Wallis test, H = 83.27, *p* < 0.01). Post hoc comparisons indicated that sharks in seagrass habitats were significantly larger, while those in muddy substrates tended to be smaller (Fig. 5).

**Fig. 6:**
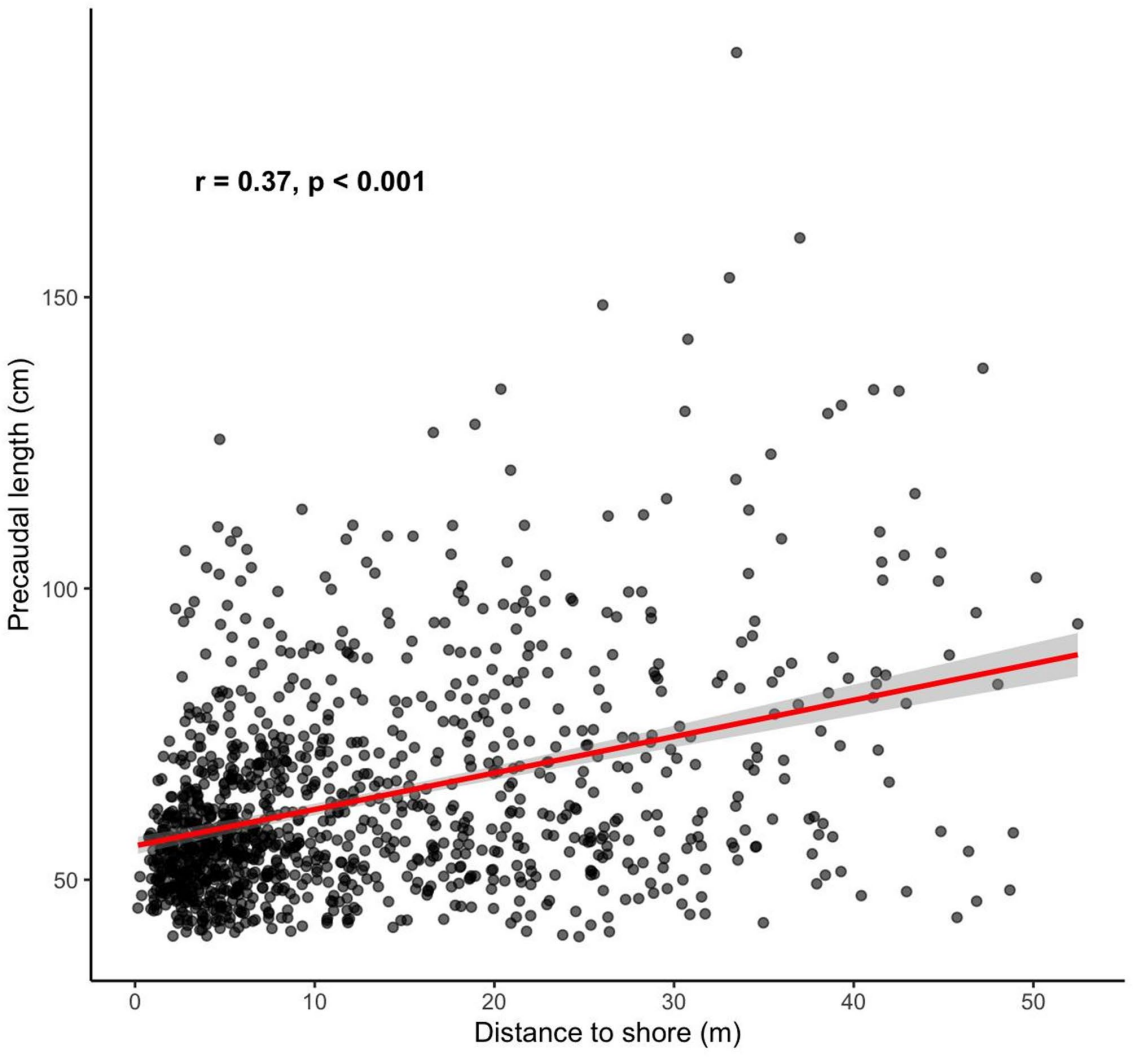
Correlation between precaudal length and offshore distance.

In addition, a significant positive correlation was found between PCL and distance from shore (*r* = 0.37, *p* < 0.001; Fig. 6), suggesting that larger individuals were more commonly observed farther offshore.

#### Environmental drivers

The best-performing model (based on AIC; Table S3) was:

$$PCL\sim Substrate+Season+Tide+Direction+(1|water.temperature+Location)$$

The model indicated that sharks were significantly larger in seagrass habitats (Estimate = 12.54, SE = 4.58, *p* < 0.05). Spring tides had a significant negative effect on PCL (Estimate = −12.15, SE = 1.63, *p* < 0.001), suggesting that sharks observed during spring tides were generally smaller (Table 2).

**Table 2:** GLMM results for PCL (Gaussian model). Random intercepts: locations, water temperature.

PCL ~ Substrate + Season + Tide + Direction + (1 | water temperature + Location)			
Fixed Effect	Estimate	Std. Error	*p* value
(Intercept)	71.72	7.76	< 0.001 ***
Season			
Winter	−2.76	2.97	0.35
Direction			
Lagoon	−4.42	6.88	0.52
North	−1.03	6.75	0.88
South	5.26	6.63	0.43
West	4.06	8.87	0.65
Tide			
Spring Tide	−12.15	1.63	< 0.001***
Substrate			
Sand	−1.60	4.54	0.72
Seagrass	12.54	4.58	0.01**

Significance: ***p < 0.001, **p < 0.01, *p < 0.05,. *p* < 0.1.

### Human activity impact on sharks

#### Shark abundance and PCL across human impact zones

**Fig. 7:**
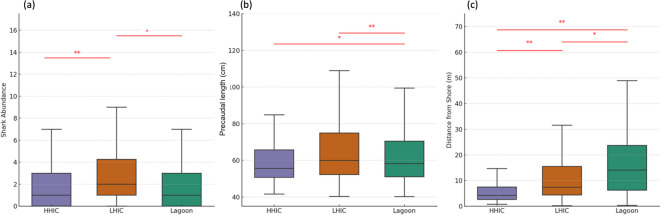
Shark (**a**) abundance, (**b**) precaudal length, (**c**) distance from shore across zones. Significance: * *p* < 0.01, **p < 0.001.

Shark abundance varied significantly among the three human activity zones (Kruskal-Wallis H = 8.00, *p* < 0.05). Post hoc comparisons indicated significantly higher abundance in LHIC compared to HHIC (U = 3129.0, *p* < 0.01), and compared to Lagoon (U = 2500, *p* < 0.05), while no significant difference was found between Lagoon and HHIC (Fig. 7a).

PCL varied significantly among the three human activity zones (Kruskal–Wallis H = 14.88, *p* < 0.001). Sharks in LHIC were significantly larger than those in HHIC (*p* < 0.001), while HHIC sharks were significantly larger than those in the Lagoon (*p* < 0.05). No significant difference was found between Lagoon and LHIC (*p* = 0.064; Fig. 7b).

#### Distance from shore and habitat segregation

Shark distance from shore varied significantly across impact zones (H = 173.34, *p* < 0.001). Individuals in HHIC areas were observed significantly closer to shore than those in both Lagoon (*p* < 0.001) and LHIC (*p* < 0.001). LHIC shark also occurred closer to shore than Lagoon individuals (*p* < 0.001, Fig. 7c).

## Discussion

This study implemented a dual-UAV strategy to survey the coastline and lagoon of Dongsha Island, enabling high-resolution, seasonal monitoring of *N.acutidens*.

This approach proved particularly effective in capturing the consistent seasonal use of Dongsha’s shallow habitat by juvenile sharks across seasons, with no significant difference in total abundance between summer and winter. Most individuals measured were below the maturity threshold of 170 cm PCL^17^, consistent with nursery habitat criteria: high juvenile density, repeated use, and site fidelity^28^. Our observations, supported by previous genetic and tagging studies, confirm the role of Dongsha Island as a nursery area for *N. acutidens*^15,19,29^.

The shift in size-frequency toward slightly larger individuals in winter suggests cohort growth. However, the concurrent presence of smaller individuals indicates possible late-season recruitment. The overlap of size classes may reflect an extended parturition period, consistent with observations of neonates as late as November^15^. The average juvenile growth rate for *N.acutidens* at Dongsha has been estimated at 0.868 cm/month^19^, which supports the interpretation that the observed seasonal size shift reflects several months of growth. This rate also aligns with the presence of slightly larger individuals during winter surveys, assuming parturition occurred in late summer or autumn.

Seasonal changes in size class distribution may also result from natural mortality or growth variation, potentially influenced by environmental stress^30,31^, food limitation^32^, or predation^33–35^.

While total abundance remained stable across seasons, our high-resolution UAV surveys revealed marked seasonal differences in fine-scale patterns across sites. Northeastern areas, including EC, ES, EN, and FB, experienced a winter decline in shark sightings. This spatial shift is likely driven by seasonal monsoonal exposure, as northeast monsoon conditions strongly influence Dongsha Island in winter^36^. During winter, northeast monsoon patterns increase wave energy and environmental instability^37^ along the eastern coastline, reducing habitat suitability for juvenile sharks.

In contrast, southern and southwestern sites such as EB, SC, Port, and SB7 exhibited significantly higher shark abundance during winter. These areas are more sheltered from prevailing winds and currents, and likely function as seasonal refuges that offer more stable foraging and resting conditions during periods of climatic stress. This redistribution aligns with previously observed seasonal habitat shifts in other reef-associated shark species^38^, and highlights the importance of local hydrodynamics in shaping shark space use.

Some sites, including NLO and SS, maintained consistently high abundance throughout both seasons. At NLO, this may be attributed to strong tidal flushing, greater depth (40–60 cm), and proximity to lagoon channels, which together support greater prey availability^15,29,36^. At SS, the presence of extensive sand flats likely provides preferred habitats for *N. acutidens*, which are known to prefer sand-flat habitats^39^.

GLMM analysis of shark abundance identified significant interaction effects between Season, Direction, Tide, and Substrate type (Chisq = 33.13, *p* < 0.005), reinforcing the role of environmental heterogeneity in structuring for capturing ecologically meaningful spatial dynamics and providing critical insight for habitat-specific conservation planning.

Beyond seasonal patterns, clear patterns of size-based spatial segregation were observed around Dongsha Island. Smaller individuals were primarily concentrated in semi-enclosed lagoon areas such as Garage and LT. These spatial trends may reflect habitat preferences linked to developmental stage, such as the selection of sheltered areas during early life. Although we cannot confirm the specific drivers of this pattern, previous research suggests that neonate and young juvenile sharks often use shallow, protected habitats that may offer refuge from predators and environmental variability^40–42^. Further studies incorporating predator abundance, physiological tolerance, and fine-scale habitat features are needed to clarify the underlying mechanisms.

Larger individuals were typically found further offshore, supporting the hypothesis of ontogenetic habitat expansion. As juvenile sharks grow, their increased mobility, energetic demands, and reduced vulnerability to predation allow them to access broader habitats and exploit new foraging opportunities^26,43,44^. This spatial separation between size classes may also reduce intraspecific competition, as observed in other coastal shark species such as *N. brevirostris*, *C. limbatus*, *Carcharhinus amboinensis*, *Sphyrna lewini*^32,33,45,46^.

Based on our GLMM results (Table S4), seasonal differences in PCL structure were influenced by tidal and thermal conditions. During summer, smaller individuals were more frequently observed during spring tides, likely exploiting shallow intertidal zones that become temporarily accessible. Such ephemeral habitats may offer increased foraging opportunities or reduced predator presence^29^. In winter, colder water temperatures (21.2 –26.3 °C) were significantly associated with smaller individuals, suggesting possible thermal habitat selection or differential mobility among size classes. Seagrass areas supported significantly larger sharks, particularly in winter, potentially due to their structural complexity, higher prey abundance, and refuge value. These findings highlight how both abiotic (e.g., temperature, tide) and biotic (e.g., substrate type, prey availability) factors interact with ontogeny to influence spatial use, and underscore the importance of protecting habitat diversity within nursery systems.

The influence of anthropogenic factors on shark distribution patterns added another layer of complexity to our findings. Contrary to expectations, shark abundance in HHIC zones was not significantly lower than in Lagoon areas, although LHIC zone consistently hosted the highest abundance. This pattern suggests that human disturbance does not entirely exclude sharks from HHIC zones, but may influence other aspects of their spatial or demographic structure.

UAV imagery revealed that sharks in HHIC were consistently observed closer to shore compared to Lagoon and LHIC. This behavior may be influenced by two factors: the attraction to anthropogenic food subsidies and the avoidance of vessel-related disturbance. Anthropogenic provisioning has been shown to influence shark behavior, increasing site fidelity, modifying movement patterns, and prolonging residency time at feeding sites across various species^47–49^. Proximity to the shoreline in HHIC zones may also reflect avoidance of deeper waters subject to frequent vessel traffic or elevated underwater noise^26,50^.

PCL distributions further underscore these patterns: LHIC zones were dominated by larger juveniles, while HHIC zones supported a higher proportion of small-bodied individuals. One possible explanation is that human disturbance may reduce habitat suitability for larger individuals, potentially leading to spatial segregation by size. While this remains a plausible explanation, further studies are needed to clarify the mechanisms underlying such demographic shifts in response to human activity.

Early-life exposure to disturbance may impair behavioral development, reduce foraging efficiency, and increase dependency on artificial food sources^32,48,50^. Over time, these effects could compromise survival, impair natural dispersal, and disrupt gene flow among nursery populations. In remote atoll systems like Dongsha, where population connectivity may already be limited, such impacts warrant urgent attention and long-term monitoring.

Our findings support previous long-term observations by Chen and Huang (2024), which identified Dongsha Island as a critical nursery habitat for *N. acutidens*—multiple zones supporting key life stages. In particular, the Lagoon (Garage, LT, NL), NLO, and EB consistently supported high shark abundance or functioned as seasonal aggregation sites. These areas should be prioritized for protection, especially during peak parturition and recruitment periods.

While our study revealed distinct spatial and temporal shifts in abundance, these results were based on a single summer and winter survey. As such, the seasonal patterns reported here should be interpreted with caution and considered preliminary.

In addition, UAV-derived PCL estimates are subject to certain measurement uncertainties. Factors such as camera angle, water surface distortion, and the absence of standardized scale references may introduce errors^27,51^. Nonetheless, studies have shown that UAV photogrammetry can yield sufficiently accurate size estimates for ecological interpretation when conducted under controlled conditions (e.g., shallow depth, clear water, near-vertical angle). In our study, all observations were made in < 1 m shallow water with high clarity and minimal wave interference, minimizing distortion. While inherent measurement limitations may influence our length estimates, they reliably capture relative differences across space and season, which offer useful indications of possible demographic and ecological structuring, supporting the utility of UAV-based observations in remote coastal systems.

UAVs offer an efficient and minimally invasive tool for detecting spatial and temporal trends in shark abundance and habitat use. Their scalability and repeatability make them ideal for monitoring remote or logistically challenging ecosystems such as Dongsha. Despite their advantages, UAVs have limitations. Environmental conditions (e.g., glare, turbidity, and weather) and behavioral factors (e.g., submerged individuals)^23,27^ may bias abundance estimates.

UAVs also cannot provide subsurface behavioral data^25^.

Integrating UAVs with complementary methods such as BRUVs, acoustic telemetry, and diver-based monitoring would enhance long-term ecological assessment. Together, these tools provide a more complete picture of shark ecology and improve conservation outcomes.

To ensure long-term population sustainability, we recommend: (1) implementing seasonal zoning to minimize disturbance in critical nursery areas, (2) continuously monitoring environmental variability and habitat connectivity using UAV and complementary methods, (3) investigating post-nursery dispersal and regional connectivity through telemetry and genetic studies, (4) and incorporating UAV-based surveys into standard monitoring frameworks for reef shark conservation.

Looking forward, climate-driven changes in hydrodynamics, temperature, and habitat structure will likely alter shark spatial ecology in the coming decades^52^. Adaptive management that incorporates environmental forecasting, real-time monitoring, and habitat-specific protection will be essential for maintaining nursery function under shifting baselines. Regional conservation strategies should also consider gene flow and dispersal corridors beyond Dongsha’s boundaries to ensure population resilience across the broader South China Sea.

By integrating ecological evidence with emerging monitoring technologies, management agencies can proactively safeguard juvenile shark populations and preserve the ecological integrity of one of the region’s most important reef systems.

## Methods

### Ethics declaration

This study involved non-invasive observation of free-ranging sharks using UAVs and did not involve any direct interaction or handling of animals. Therefore, no animal ethics approval was required. All observational protocols were conducted in accordance with relevant guidelines and regulations.

### Survey design, parameters, and calibration

UAV surveys were conducted during summer (August 2021) and winter (February 2022). Each flight systematically covered the nearshore waters surrounding Dongsha Island, targeting shark abundance, PCL, and spatial distribution. All surveys were timed within 30 min of high tide to maximize the visibility of sharks in shallow waters (< 1 m). Two trained UAV pilots flew simultaneous clockwise and counterclockwise missions from elevated land-based positions on Dongsha Island to achieve complete shoreline coverage.

Flight paths were planned using QGIS 3.28.2^53^, referencing Google satellite imagery. A 17 m offset from shoreline was applied to optimize in-water visibility, and coordinates were projected to EPSG: 32,650 (WGS 84/ UTM Zone 50 N) to ensure accurate spatial referencing. The total flight path length was 11.5 km, which was manually split into two equal-length north and south segments (5.76 km each) for concurrent operations. These routes were exported as KML files and uploaded into UgCS v.4.4 (https://www.ugcs.com/) mission-planning software for automated flight execution.

Surveys were conducted using Autel EVO II 6k (Autel Robotics Co., Ltd., Seattle, USA), equipped with a 6 K-capable camera (5760 × 3240 pixels). Flights were conducted at a fixed altitude of 47 m, providing a frame coverage of approximately 54 m × 30.3 m. To optimize data processing efficiency while maintaining sufficient resolution for shark detection and measurement, videos were recorded at 2.7 K (2720 × 1528).

To ensure accurate size estimation from UAV footage, image scaling was calibrated under field conditions. A 1-meter reference scale was submerged at a depth of 1 m and filmed using the UAV system. Analysis of the footage determined a consistent spatial resolution of 0.356 pixel per centimeter, indicating that 100 cm in real-world distance corresponded to 35.63 pixels in the image. The footage was analyzed in ImageJ 1.53p (https://imagej.net/ij/).

As all survey areas consistently had water depths less than 1 m, vertical distortion due to depth-related refraction was minimized, enhancing the reliability of UAV-based size estimates for relative spatial and seasonal comparisons.

The calibration method has been adopted in UAV-based studies of marine megafauna, where in-water scale bars or objects of known length are used to correct spatial distortion and enable reliable measurements. For instance, Whitehead et al. (2022) applied a similar method using 1-meter scale bars to estimate whale shark PCL, achieving < 2% error under optimal visibility and depth conditions^54^. Comparable approaches have been used in sea turtle studies, even under greater depth and refraction variables, to estimate small anatomical features such as tail length^55,56^. These studies support the validity of our calibration method, especially given the shallow (< 1 m), calm water conditions in Dongsha.

###  Statistical analysis

#### Abundance

Following each UAV survey, two trained observers independently reviewed footage to count all visible sharks. To evaluate the influence of multiple environmental variables including Season (Summer vs. Winter), Tide (Spring vs. Neap), Direction (North, East, South, West, Lagoon), Substrate type (Seagrass, Sand, Mud), and Water Temperature, we used Generalized Linear Mixed Models (GLMMs) implemented via the ‘glmmTMB’ package^57^ in R v4.2.1^58^. Interaction terms (e.g., Season × Tide, Tide × Substrate) were tested to assess potential context-dependent effects.

Given that shark abundance is count-based, we tested for overdispersion to select the appropriate error structure (Poisson or Negative Binomial). Random effects were included to account for repeated surveys at each location (e.g., 1 + Season | Location), allowing environmental effects to vary across sites.

We adopted an information-theoretic approach using Akaike’s Information Criterion (AIC) to identify the most parsimonious model that best explains shark abundance in relation to ecological and environmental factors^59^. Residual diagnostics, overdispersion, and zero-inflation were assessed using ‘DHARMa’ package^60^ to ensure appropriate model fit and validity. Initial diagnostics revealed overdispersion under a Poisson distribution (dispersion ratio = 1.98, *p* = 0.144), supporting a Negative Binomial error structure. Although zero-inflation was statistically significant (*p* < 0.001), the observed-to-simulated zero ratio (0.03) was minimal, and inclusion of a zero-inflation term did not improve model performance based on AIC. Therefore, a standard Negative Binomial GLMM was selected as the most parsimonious model.

#### PCL estimation and spatial distribution

To estimate shark body size from aerial imagery, we measured PCL instead of total length to reduce potential error caused by caudal fin movement, body curvature, or water surface refraction, using ImageJ (Fig. S3).

PCL distributions were compared across seasons and locations using Mann-Whitney U tests. Differences among substrate types were assessed via Kruskal-Wallis tests with Dunn’s post hoc tests. To visualize seasonal variation in size structure, we generated kernel density estimates (KDEs).

To evaluate spatial trends in size, we conducted Pearson’s correlation analysis between shark size and distance from shore, testing whether larger individuals were more likely to occur offshore. Pearson’s correlation tests were performed using the cor.test() function, which provides both the correlation coefficient (r) and the significance level (p-value).

While non-parametric tests revealed pairwise differences in shark size across seasons and substrates, these approaches could not account for potential interactions or random site-level variation. To assess the combined influence of multiple environmental variables while controlling for spatial and thermal heterogeneity, we applied GLMMs (Gaussian error structure) to assess the effects of ecological variables (Season, Tide, Water Temperature, Substrate, and Direction) on PCL variation, with Location included as a random effect. Model selection was based on AIC, and residual assumptions were validated using the ‘DHARMa’ package.

### Human impact zones and ecological comparisons

To evaluate anthropogenic effects on shark distribution, we categorized the 20 observation sties into three habitat types based on disturbance level: (1) Lagoon: Enclosed shallow area with mixed substrates (seagrass, sand, mud) and minimal disturbance (NL, LT, Garage, SL, SLO, SS); (2) LHIC: Nearshore habitats with limited human activity (NC, NW, NLO, EB, ST, SC); and (3) HHIC: Sites adjacent to infrastructure or human settlements and frequently exposed to anthropogenic activity (SB2, EN, EC, FB, ES, Port, SB6, SB7). Classification was based on proximity to areas of vessel traffic, the presence of infrastructure (e.g., patrol docks, research station, military quarters), and direct observations of human activity.

We compared shark abundance, PCL, and distance from shore across these zones using Kruskal-Wallis tests with Dunn’s post hoc tests where applicable. All statistical analyses were performed in R v4.2.1^58^.

## Supplementary Information

Below is the link to the electronic supplementary material.

Supplementary Material 1

## Data Availability

All data supporting the findings of this study, including UAV-based shark sightings, size measurements, and environmental variables, are included in this article and its supplementary materials.
