# Supplementary material for: Monitoring juvenile sicklefin lemon shark Negaprion acutidens in remote marine nurseries using unmanned aerial vehicles (UAVs)
Source: Sci Rep. 2025 Oct 24;15:37242. doi: 10.1038/s41598-025-21142-y (PMC12552443; doi:10.1038/s41598-025-21142-y)
Supplement: Supplementary file 1 — Supplementary Material 1 [file 41598_2025_21142_MOESM1_ESM.docx]

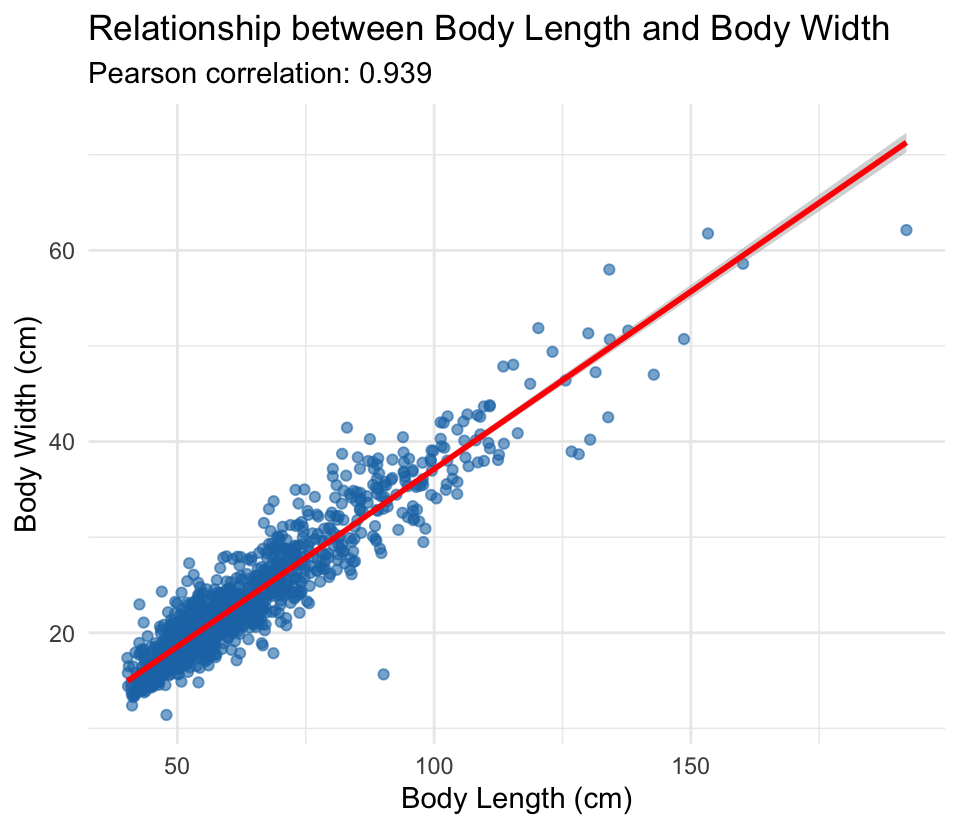


Fig. S1. Relationship between Body Length (PCL) and body width of observed *N. acutidens.* A strong positive correlation was found between body length and body width (Pearson’s r = 0.939), indicating a high degree of collinearity between the two size metrics. Given this strong association, body length was used as the sole proxy for overall body size in subsequent analyses.


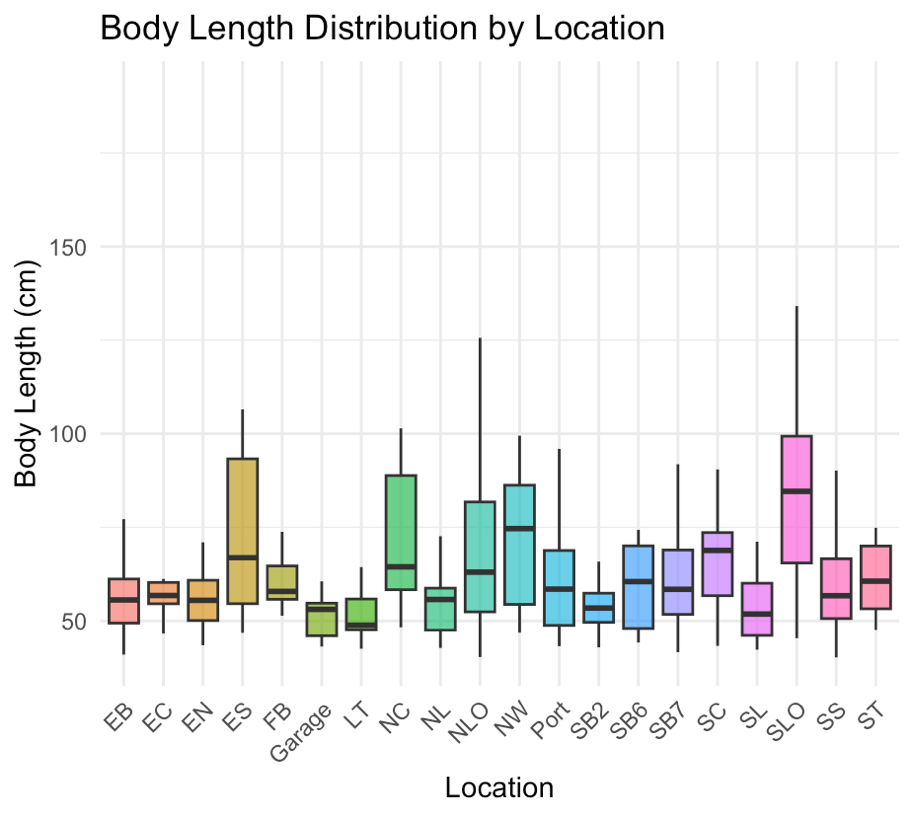


Fig. S2. Shark body length (PCL) across sites (all seasons combined)


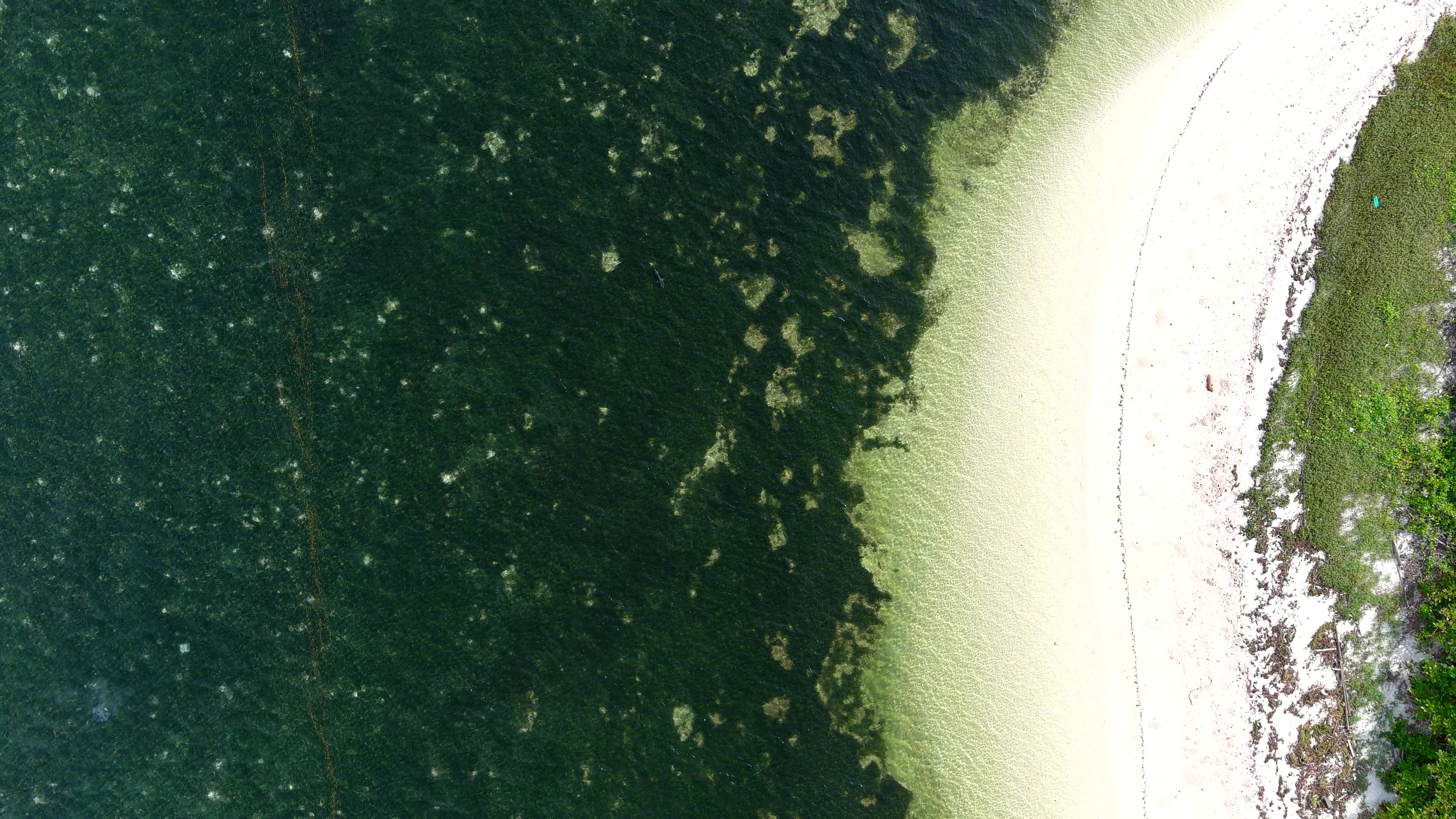

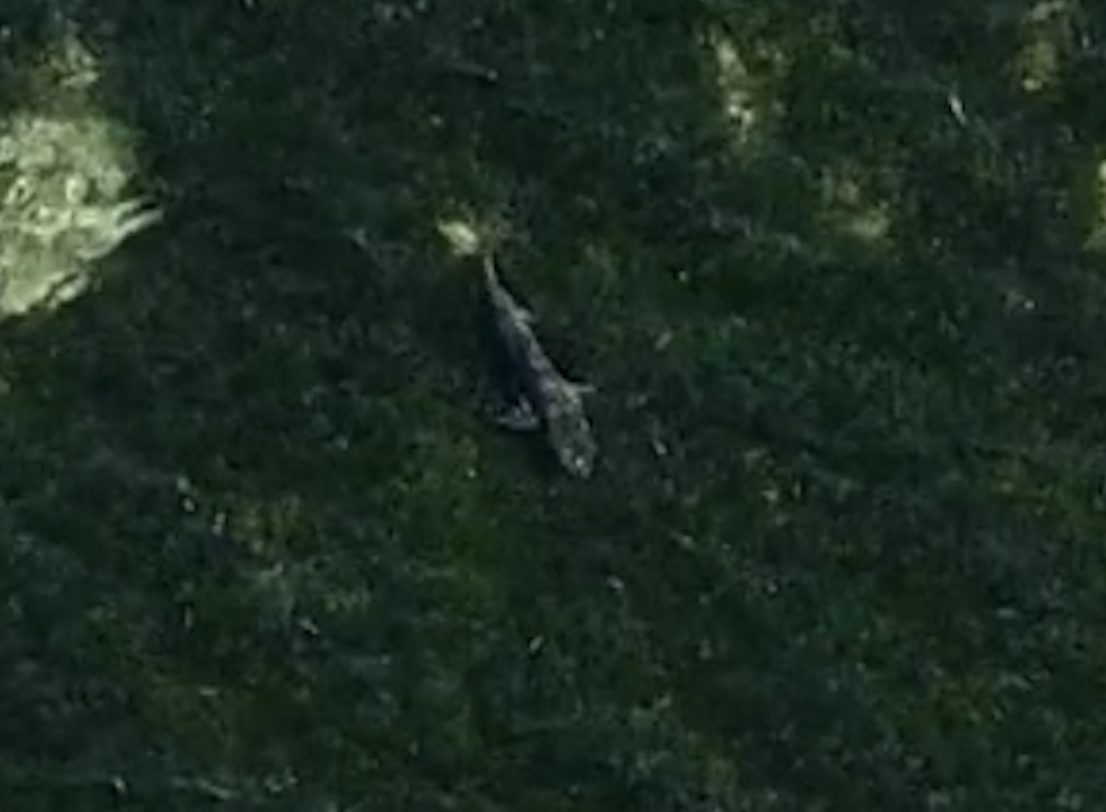


Fig. S3. Example of UAV imagery showing a *N.acutidens* detected in shallow nearshore waters around Dongsha Island. This inset highlights a single shark individuals, with a yellow reference line indicating the measured PCL

Table S1. Summary of shark counts by survey date and season

| Summer | | Winter | |
| --- | --- | --- | --- |
| Date | Total shark count | Date | Total shark count |
| 2021/8/12 | 145 | 2022/2/7 | 41 |
| 2021/8/14 | 63 | 2022/2/8 | 60 |
| 2021/8/15 | 56 | 2022/2/9 | 45 |
| 2021/8/22 | 116 | 2022/2/10 | 69 |
| 2021/8/23 | 157 | 2022/2/11 | 83 |
| 2021/8/24 | 123 | 2022/2/12 | 110 |
|  |  | 2022/2/13 | 93 |
| Mean | 110 |  | 71.57 |
| SE | 17.09 |  | 9.57 |

Table S2. Akaike Information Criterion (AIC) values of candidate GLMMs for shark abundance, comparing models with different combinations of fixed and random effects

|  | df | AIC |
| --- | --- | --- |
| Season +Direction+ Tide + Water.Temperature+Substrate + (1 \| Location) | 12 | 1025.91 |
| Tide +Direction+ Water.Temperature+Substrate + (1 \| Location) | 11 | 1028.28 |
| Season +Direction + Water.Temperature+Substrate + (1 \| Location) | 11 | 1025.97 |
| Season +Direction+ Tide +Substrate + (1 \| Location) | 11 | 1025.10 |
| Season +Direction+ Tide + Water.Temperature + (1 \| Location) | 10 | 1322.67 |
| Season * Tide +Direction+ Water.Temperature+Substrate + (1 \| Location) | 14 | 1033.36 |
| Season * Water.Temperature + Direction+Tide+Substrate + (1 \| Location) | 13 | 1027.84 |
| Season + Direction +Water.Temperature+Tide *Substrate + (1 \| Location) | 14 | 1027.66 |
| Season * Substrate + Direction+Water.Temperature +Tide +(1 \| Location) | 14 | 1029.08 |
| Season + Direction *Water.Temperature+Tide+Substrate + (1 \| Location) | 19 | 1017.73 |
| Season * Direction +Water.Temperature+Tide+Substrate + (1 \| Location) | 19 | 1011.23 |
| Season * Direction + Tide + Substrate + (1 \| Location) | 15 | 1000.06 |
| Season + Direction+ Water.Temperature+Substrate +Tide * (1 \| Location) | 12 | 1025.91 |
| Tide + Water.Temperature + Direction + Substrate + (1 + Season \| Location) | 13 | 1014.12 |
| Season + Tide + Direction + Substrate + (1 + Water.Temperature \| Location) | 13 | 1017.45 |

Table S3. AIC scores and model structures of GLMMs for PCL across seasons, including interaction terms and random slopes.

|  | df | AIC |
| --- | --- | --- |
| Water temperature + Season + Substrate + Tide + Direction + (1\|Location) | 12 | 9502.88 |
| Season + Substrate + Tide + Direction + (1\|Location) | 11 | 9531.457 |
| Water temperature + Substrate + Tide + Direction + (1\|Location) | 11 | 9538.92 |
| Water temperature + Season + Substrate + Direction + (1\|Location) | 11 | 9612.258 |
| Water temperature + Season + Substrate + Tide + (1\|Location) | 8 | 9496.869 |
| Water.temperature * Substrate + Direction + (1 \| Location) | 12 | 9649.491 |
| Water.temperature * Substrate + Season + Tide + Direction + (1\| Location) | 14 | 9506.815 |
| Water.temperature * Direction + Substrate + Season + Tide + (1 \| Location) | 16 | 9463.48 |
| Water.temperature + Substrate + Season * Tide + Direction + (1 \| Location) | 13 | 9480.595 |
| Water.temperature + Direction * Season + Tide + Substrate + (1 \| Location) | 16 | 9454.315 |
| Water.temperature + Substrate * Season + Tide + Direction + (1 \| Location) | 14 | 9506.691 |
| Water.temperature + Direction * Tide + Season + Substrate + (1 \| Location) | 16 | 9448.889 |
| Substrate + Season + Tide + Direction + (1 \| water.temperature + Location) | 12 | 9413.039 |
| Water.temperature + Substrate + Season + Tide + Direction + (1 \| Season + Location) | 14 | 9510.85 |

Table S4. Summary of GLMM results for environmental predictors of PCL in summer and winter

| Variable | Summer | | | | Winter | | | |
| --- | --- | --- | --- | --- | --- | --- | --- | --- |
|  | Estimate | SE | z | p-value | Estimate | SE | z | p-value |
| Intercept | 66.963 | 9.986 | 6.706 | <0.001 *** | 122.542 | 18.395 | 6.662 | <0.001 *** |
| Spring Tide | -14.838 | 2.760 | -5.376 | <0.001 *** | -1.544 | 5.578 | -0.277 | 0.782 |
| Substrate sand | -2.707 | 7.433 | -0.364 | 0.716 | 2.589 | 6.117 | 0.423 | 0.672 |
| Substrate seagrass | 11.450 | 7.450 | 1.537 | 0.124 | 27.605 | 6.564 | 4.205 | <0.001 *** |
| Direction Lagoon | 7.155 | 7.420 | 0.964 | 0.335 | -28.674 | 9.750 | -2.941 | 0.003 ** |
| Direction North | -3.769 | 6.693 | -0.563 | 0.573 | -18.926 | 9.823 | -1.927 | 0.054 . |
| Direction South | 19.028 | 6.962 | 2.733 | 0.006 ** | -26.976 | 9.591 | -2.813 | 0.005 ** |
| Direction West | 10.922 | 9.131 | 1.196 | 0.232 | -22.744 | 10.316 | -2.205 | 0.027 * |
| Water temperature | — | — | — | — | -1.650 | 0.616 | -2.678 | 0.007 ** |
| Spring Tide × sand | — | — | — | — | -0.330 | 5.766 | -0.057 | 0.954 |
| Spring Tide × seagrass | — | — | — | — | -17.911 | 6.316 | -2.836 | 0.005 ** |
